# Zhibaidihuang Decoction Ameliorates Cell Oxidative Stress by Regulating the Keap1-Nrf2-ARE Signalling Pathway

**DOI:** 10.1155/2020/9294605

**Published:** 2020-02-12

**Authors:** Pingping Wu, Bin Ding, Li Ye, Yanfen Huang, Jinjun Ji, Yongsheng Fan, Li Xu

**Affiliations:** ^1^College of Basic Medicine, Zhejiang Chinese Medical University, Hangzhou, Zhejiang, China; ^2^College of Life Science, Zhejiang Chinese Medical University, Hangzhou, Zhejiang, China; ^3^First Clinical Medical College, Zhejiang Chinese Medical University, Hangzhou, Zhejiang, China

## Abstract

Zhibaidihuang decoction (ZBDHD) is a Chinese herbal formula, which is used in Chinese traditional medicine to treat symptoms of Yinxuhuowang (Yin deficiency and high fire) syndrome. This study elucidates the mechanism of ZBDHD on oral ulcers, one Yinxuhuowang syndrome. Simultaneously, some ingredients in ZBDHD were found and identified by ultraperformance liquid chromatography-tandem mass spectrometry (UPLC-MS/MS). A Ganjiangfuzirougui decoction- (GJD-) induced Yinxuhuowang syndrome SD rat model was used to demonstrate the efficiency of ZBDHD treatment. The oral mucosa of rat in the GJD group, stained with hematoxylin and eosin (H&E), showed epidermal shedding and inflammatory cell infiltration. And an alleviation efficiency of ZBDHD in GJD-induced pathological changes in the oral mucosa could be obtained. ZBDHD treatment restored the GJD-induced imbalance of metabolites, which were choline, glycocholic acid, and palmitoyl-L-carnitine (PALC). GJD stimulated the expression of NF-*κ*B. And the overexpressed of NF-*κ*B in mucosa of rat in the GJD group could be inhibited by ZBDHD treatment. Simultaneously, the optimal efficiency of ZBDHD treatment on the cellular ATP content, oxygen consumption rate (OCR), and superoxide dismutase (SOD) concentration was evaluated, in vitro assay. Compared to the control cells, the ATP content, OCR, and SOD activity in the ZBDHD-treated cells were significantly higher. For the mechanisms study, seven cytokines were screened with a Dual-Luciferase Reporter gene assay. In the ARE assay, the luciferase signal was stimulated significantly by ZBDHD. In cells, the transcription of *nrf2*, *maf*, and *keap1*, which were related to the ARE pathway, was elevated by ZBDHD treatment. Our study demonstrated that high-dose GJD could lead to Yinxuhuowang syndrome, such as oral ulcers, and the imbalance in serum metabolites. And ZBDHD can improve oral cell inflammation and the imbalance of metabolism by inhibiting NF-*κ*B and enhancing the activity of the ARE signalling pathway to ameliorate oxidative stress in the cell. This study provides a theoretical basis for the clinical application of ZBDHD.

## 1. Introduction

For all living aerobic creatures, molecular oxygen is the ultimate electron acceptor for cellular respiration. Reactive oxygen species (ROS) are generated during oxidative phosphorylation in mitochondria [1]. The first intermediate in the oxygen reduction process consists of superoxide radicals (O_2_^.-^), which are rapidly converted to hydrogen peroxide (H_2_O_2_) [2]. H_2_O_2_ can also be generated deliberately during phagocytosis in macrophages and neutrophils [3]. Simultaneously, ROS can be exogenously stimulated, e.g., through cosmic radiation or UV light [4]. The term “oxidative stress” implies a physiological imbalance in the ROS creation and scavenging. Inflammation, which is triggered by oxidative stress, is associated with many diseases, such as oral ulcers [5], neurodegenerative diseases [6], tumour [7, 8], cardiovascular disease [9, 10], type 2 diabetes [11], nonalcoholic fatty liver disease [12], and Parkinson's disease [13]. Since it has been recognized that antioxidants can alleviate inflammatory reactions, some antioxidants have been studied and proposed for therapeutic use based on their therapeutic effects [14], e.g., vitamin C, coenzyme Q10, and some polyphenols that are isolated from herbs [15].

People from China, as well as those from some other ancient cultures, such as those in India and Africa, have continuously used their traditional alternative medicines. Some well-studied therapeutic compounds, such as artemisinin [16] and soy isoflavones [17], were derived from traditional Chinese herbal medicines. However, these medicinal herbs are generally administered by prescriptions. In traditional Chinese medicine (TCM), health is defined as a balance between Yin (cold) and Yang (hot, fire) [18]. Yin and Yang syndrome types reflect the two sides of the TCM concept. TCM doctors treat patients mainly based on syndrome differentiation. Generally, the TCM doctor uses cold (Yin-enriched) herbal prescriptions to treat Yang syndrome patients and hot prescriptions to treat Yin syndrome patients.

As noted in the “Yi Zong Jin Jian” (an ancient book about traditional Chinese medicine) [19], ZBDHD is one of the prescriptions developed from Liuweidihuang (LWDH) on the basis of an original formulation to which the *Anemarrhenae* and *Cortex Phellodendri* are added. Clinical research has shown that it has hypoglycaemic effects, confers enhanced immunity, has antioxidant and antifatigue properties, and regulates neuroendocrine and antitumour efficiency [20]. In comparison with the original LWDH, ZBDHD enhances the ability to tonify Yin as well as clear “hot” according to TCM theory [20]. In addition, it can be used clinically to relieve numerous diseases, such as various syndromes of menopausal [21], polycystic ovary syndrome hyperandrogenism [22], and recurrent oral ulcers [23], which are associated with “Yin deficiency and high fire” (Yinxuhuowang syndrome) [24]. In this study, we evaluated the “tonifying Yin and clearing hot” capability of ZBDHD with a Yinxuhuowang syndrome rat model [25].

## 2. Materials and Methods

### 2.1. Chemical and Biochemical Materials

DMEM (E9013), fetal bovine serum (12250), and RPMI1640 cell culture medium (E9020) were purchased from GENOM (Hangzhou, China). The EndoFree Plasmid Maxi kit (DP117) and annealing buffer for DNA oligos (5X) (D0251) were purchased from TIANGEN (Beijing, China) and Beyotime Biotechnology (Shanghai, China), respectively. The plasmids for Dual-Luciferase Reporter Assay tests, pHIF-1-Luc (GM-021020), pE2F-Luc (GM-021046), and pSRE-Luc (GM-021086) were obtained from Genomeditech (Shang

hai, China). And pISRE-Luc (D2152), pP53-TA-Luciferase (D2223), and pARE-Luciferase (D2112) were obtained from Beyotime Biotechnology (Shanghai, China); pSIE-Luc was obtained from the Lab of Prof. Qin (Shanghai University of Traditional Chinese Medicine). The pRL Renilla Luciferase Control Reporter Vector (E2231) and the Dual-Luciferase® reporter assay system (E1910) were obtained from Promega (Madison, USA). dNTP (4019), recombinant RNase inhibitor (2313A), reverse transcriptase M-MLV (2641A), and TB Green (RR420A) were all purchased from TaKaRa (Dalian, China). The primers used in this study were synthesized by Bioengineering (Shanghai, China). The ATP assay kit (A095-1-1), reactive oxygen species (ROS) test box (E004), and superoxide dismutase (SOD) test box (E001-3) were obtained from Jiancheng (Nanjing, China). The MitoXpress Xtra Oxygen consumption assay kit (MX-200) was from Agilent Technologies, Inc. (Agilent, Santa Clara, CA, USA). SuperFectinTM II In Vitro DNA Transfection Reagent (2102-100) was obtained from Pufei Biotech (Shanghai, China). *β*-Actin (C4) (sc-47778) was obtained from Santa Cruz (Delaware, USA), and NF-*κ*B-P65 Monoclonal Antibody (YM0474) was obtained from ImmunoWay (Plano, USA). Antimycin (A8674) and FCCP (C2920) were purchased from Sigma-Aldrich (Missouri, USA).

### 2.2. Equipment

A direct heat CO_2_ incubator (Forma™ 311), Varioskan® Flash Microplate Multimode Readers, and NanoDrop One UV-Vis spectrophotometer were purchased from Thermo Fisher Scientific (Waltham, MA, USA). A LightCycler PCP was purchased from Roche (CA, USA). A Centrifuge 5417R was obtained from Eppendorf (Hamburg, Germany). Agilent 1260 HPLC/6520 QTOF-MS instrument, Eclipse Plus C18 chromatography column, and Cary Eclipse fluorescence spectrophotometer were obtained from Agilent Technologies, Inc. (Agilent, Milford, MA, USA). Ultra-High-Performance Liquid Chromatography System and ACQUITY UPLC HSS C18 column (100 × 2.1 mm, 1.7 *μ*m) were from Waters (Milford, MA, USA). The X500 QTOF Mass spectrometer system was purchased from AB Sciex Pte. Ltd. (MA, USA).

### 2.3. Herbal Extract and Lyophilized Powder Preparation

The ingredients in the ZBDHD formula were 24 g Huangbai (cortex *Phellodendron chinense* Schneid.), 24 g Zhimu (*Anemarrhena asphodeloides* Bge.), 24 g Shudihuang (Radix *Rehmannia glutinosa* Libosch. Preparata), 12 g Shanzhuyu (*Cornus officinalis* Sieb. et), 12 g Shanyao (radix *Dioscorea opposita* Thunb.), 9 g Zexie (*Rhizoma Alisma orientalis*), 9 g Fuling (Sclerotium of *Poria cocos* (Schw.) Wolf), and 9 g Mudanpi (*Paeonia suffruticosa* Andr.). The GJD formula comprised 200 g Gan Jiang (Rhizoma Zingiberis), 200 g Dan Fu Zi (Daughter Root of *Aconitum Carmichaelii Debx.*), and 200 g Rougui (*Cinnamomum cassia*). These herbs were obtained from the Zhejiang Chinese Medical University Medical Slicing Factory, Ltd. (Hangzhou, China). The ZBDHD and GJD decoctions were prepared following the traditional method, separately; the herbs were soaked in cold water for 30 min (min) and boiled in water for 30 min. Finally, one-half of the decoction was condensed to 1 g/mL (1 g herbal mixture per mL) by a R202 rotary evaporator for animal experiment. And the other half of each sample was freeze-dried.

### 2.4. UPLC-MS/MS Analysis

4 *μ*L 4 mg/mL ZBDHD decoctions was loaded into an ACQUITY UPLC HSS C18 column (100 × 2.1 mm, 1.7 *μ*m) by an Ultra-High-Performance Liquid Chromatography System. And the system was operated at a flow rate of 0.3 mL·min^−1^. The eluate-mobile phase comprised water with 0.1% formic acid (solution A): acetonitrile (solution B) in a gradient mode as follows: 0–2 min, 95% B; 2–17 min, 95% B; 17–20 min, 45% B; 20-21 min, 5% B; 21–24 min, 5% B; and 13.1–15 min, 5% B. The injected sample volume was 4 *μ*L. The temperatures of column and autosampler were maintained at 40°C and 8°C, respectively. The MS data were acquired with electrospray ionization mass spectrometer-mass spectrometry (ESI-MS/MS), in both positive and negative ion mode, with X500 QTOF mass spectrometer system. The dual ion source was operated in negative ion ESI mode following evaluation of analyte sensitivity and noise backgrounds observed in both positive and negative ion modes. The MA parameters were as follows: Ion Source Gas1 (Gas1): 55, Ion Source Gas2 (Gas2): 55, Curtain gas (CUR): 35, source temperature: 600°C, Ion Sapary Voltage Floating (ISVF)-4500 V, and TOF MS scan *m*/*z* range: 50–1500 Da.

### 2.5. Animal Treatment

Forty-five Sprague-Dawley (SD) female rats, 6–8 weeks old and weighing 200 ± 20 g, were purchased from the animal experimental center of Zhejiang Chinese Medical University. All animal experiments in this project have passed the ethics committee of Zhejiang Chinese Medical University Animal Research Center (Accepted Nr. ZSLL-2016-116). The rats were separately handled under specific pathogen-free (SPF) conditions under a strict light cycle (12 h of light) at a temperature of 20°C and relative humidity of 40%–60%. For the study, the rats were randomly separated into 3 groups with 15 rats in each group: the control group (Ct) (intragastric administration of 0.1 mL 0.9% saline solution per 10 g body weight per day for 21 days); the GJD group (intragastric administration of 0.1 mL GJD per 10 g body weight per day for 14 days, and 0.1 mL 0.9% saline solution for 7 days); and the GZ group (intragastric administration of 0.1 mL GJD per 10 g body weight per day after 14 days, followed by intragastric administration of 0.1 mL ZBDHD for 7 days). All rats in the three groups had free access to water and a general diet. On the 21st day, the rats were anaesthetized with an intraperitoneal injection of 5% chloral hydrate solution (3.3 mL/kg). The blood, which was collected from the abdominal vein of rats, was maintained at rest for 30 min to precipitate the cells in serum. After 3000 rpm centrifugation at 4°C for 10 min, the serum was pipetted into a 5 mL tube and preserved at −80°C for subsequent cytokine testing and serum metabolomics research. The oral mucosa of the rats was fixed with 4% paraformaldehyde solution and embedded in paraffin. This paraffin-sealed tissue was cut into thin 4 *μ*m slices and stained with hematoxylin and eosin (H&E) for histopathological observation.

### 2.6. Immunohistochemistry Detection

The paraffin-embedded sample was cut into thin sections (4 or 5 *μ*m) and sealed in 3% H_2_O_2_ at room temperature to inactivate enzymes. Then, the sections were boiled in 10 mM sodium citrate buffer (pH 6.0) for 10 min and cooled at room temperature. For immunohistochemistry analysis, the sections were blocked with normal goat serum and then hybridized with primary antibodies against NF-*κ*B (1 : 200) overnight at 4°C. The primary antibodies were detected with fluorescence-labelled corresponding secondary antibody. Simultaneously, DAPI staining was performed to stain the nuclei. More details were described in previous study [26].

### 2.7. Metabolomic Analysis by HPLC-QTOF/MS

High-performance liquid chromatography (HPLC) coupled to electrospray-ionization MS (ESI/MS) was used for metabolite identification and quantitation in this study. The serum pretreatment process was completed as follows. At room temperature, 150 *μ*L serum was well mixed with 450 *μ*L acetonitrile with a vortex mixer for 30 s. This mixture was incubated at room temperature for 10 min. The denatured protein was precipitated by high speed (at 4°C, 12000 rpm for 10 min). And the 300 *μ*L supernatant was mixed with an equal volume of deionized water and filtered with a 0.22 *μ*m sterile nylon membrane. For HPLC-ESI/MS, 5 *μ*L of pretreated serum sample was loaded onto an Eclipse Plus C18 chromatographic column (2.1 × 50 mm, 1.8 *μ*m) for HPLC. An elution gradient programme was run for chromatographic separation with mobile phase A (0.1% formic acid in aqueous solution) and mobile phase B (0.1% formic acid in acetonitrile solution) as follows: 0 min, 95% A and 5% B; 4 min, 90% A and 10% B; 17 min, 55% A and 45% B; and 22 min, 5% A and 95% B. The flow rate was 0.2 mL/min; the column temperature was 35°C; and the post time was 5 min. The ESI mass spectra for sample analysis were acquired in positive ion mode (ESI+). The optimal operating parameters were similar to the following: acquisition mass range, *m*/*z* 50∼1000; drying gas, N_2_; flow rate, 10 L/min; evaporation temperature, 350°C; capillary, 4000 V; breaking voltage, 180 V; cone hole voltage, 60 V; and atomization pressure, 310 kPa. The mass spectrometry data were collected as 2 spectra/s.

### 2.8. Growth of 293T Cells

293T cells was obtained from Shanghai Cell Bank of the Chinese Academy of Sciences (Shanghai, China) and grown with DMEM containing 10% fetal bovine serum at 37°C in a 5% CO_2_ incubator in a cell culture flask until the cells are 85–90% confluent. The log phase grown cells were cultured in a 48-well microtiter plate (10^4^ cells/well) for 24 h, for ZBDHD (0, 1, 20, and 100 mg/mL) treatment or other experiments.

### 2.9. Quantitative Analysis of ATP

The concentration of ATP in 293T cells was quantified by a colorimetric assay kit. A total of 1 × 10^6^ cells were resuspended in 300 *μ*L ddH_2_O and ultrasonically lysed in a hot water bath and centrifuged to obtain the cell extract. A total of 30 *μ*L cell extract was mixed with 100 *μ*L substrate buffer I, 200 *μ*L substrate buffer II, and 30 *μ*L promoting buffer and incubated at 37°C for 30 min. In this assay, the ATP in cells and the creatine in the substrate buffer catalysed phosphocreatine synthesis. The concentration of phosphocreatine was quantified with molybdenum blue reagent, which was determined at 636 nm. The ATP concentration was calculated with respect to the standard ATP solution and the negative control assay.

### 2.10. Determination of the Oxygen Consumption Rate

The oxygen consumption rate in cells was determined with 2,7-dichlorodihydrofluorescein diacetate (DCFH-DA) reagent. In accordance with the instructions in the kit, 10 *μ*L of the probe solution, supplied with the kit, was added to the 10^6^ ZBDHD treated 293T cells. In one hour, the cells were washed and resuspended in 200 *μ*L PBS. Oxidation of the DCFH converted the molecule into its fluorescent form, which was used for detection by a fluorescence spectrophotometer at an excitation wavelength of 485 nm and an emission of 525 nm.

### 2.11. Evaluation of SOD Activity

The SOD concentration in 293T cells was evaluated with a colorimetric assay kit. A total of 10^6^ cells were resuspended in 300 *μ*L ddH_2_O and ultrasonically lysed in an ice bath. In addition, 20 *μ*L cell lysate was mixed with enzyme buffer and the substrate solution supplied in the kit. After incubation at 37°C for 20 min, the absorption of each test sample was evaluated at 450 nm. The SOD activity was calculated in accordance with the instructions in the kit.

### 2.12. Signal Pathway Detection by Dual-Luciferase Reporter Assay

The following seven cytokines were examined in this study: antioxidant response element (ARE), hypoxia-inducible factor (HIF-1), cell cycle-related transcription factor response element (E2F), promoter tumour suppressor protein 53 (pP53), interferon-stimulated response element (ISRE), serum response element (SRE), sis-inducible element, and sis-inducible reaction element (SIE). The plasmids were separately transformed into *E. coli* DH5*α* for amplification and then were isolated from *E. coli* strains, respectively, according to the protocol of the EndoFree Plasmid Maxi kit, respectively. The 293T cells were grown in 48-well plates and transfected with plasmids for 24 h, separately. These transfected cells were treated with different concentrations of GJD (0, 1, 10, 20, and 50 mg/mL) or ZBDHD (1, 20, and 100 mg/mL) for 24 h. Then, the cells in each well were lysed, and the expression of the gene in the plasmid was detected with Luciferase Reporter assays (according to the instructions in the kit). The fluorescence signal was detected by a microplate reader. Each concentration of every decoction was repeated in sextuplicate.

### 2.13. Quantitation of mRNA by Real-Time Reverse Transcriptase PCR Assay

Total RNA was extracted from ZBDHD (0, 1, 20, and 100 mg/mL) treated cells and reverse-transcribed into cDNA for PCR. As recommended, the PCR condition was as follows: denaturation at 95°C for 10 min and 45 amplification cycles of 95°C for 10 s, 55°C for 30 s, and 72°C for 10 s. The melting curve was analysed as the temperature increased from 65°C to 97°C. The sequences of the primers in this study are shown in Table 1. The PCR assay contained 1× SYBR green PCR master mix, template 100 ng, and 200 *μ*mol primer pairs.

### 2.14. Western Blot

Oral mucosa lysate was used to examine the expressions of NF-*κ*B. The protein concentration was determined by a Q5000 UV-vis Spectrophotometer. An equal amount of 20–30 *μ*g of protein was separated by 10% sodium dodecyl sulfate-polyacrylamide gel (SDS-PAGE) and transferred to polyvinylidene fluoride (PVDF) membranes by electroblotting equipment. Nonspecific protein binding was blocked with 1% bovine serum albumin in TBS with 0.1% Tween 20 (pH 7.6, 3.03 g Tris base, 18.8 g glycine, 1 g SDS, 1000 ml ddH_2_O, plus 1 ml Tween-20) for 1 h at room temperature. Then, the membranes were incubated overnight at 4°C with the diluted primary antibodies. After washing with TBST three times, the membrane was incubated with the corresponding HRP-conjugated secondary antibody (1 : 2000) at room temperature for 1 hour and then washed for three more times with TBST. Protein bands were visualized using the enhanced chemiluminescence detection system (Boster ECL Reagent kit). The experiments were repeated three times independently. A monoclonal antibody specific to *β*-actin (1 : 500) was used to determine the equal protein loading.

### 2.15. Statistical Analysis of Data

All data were expressed as means (+) standard deviation ($\bar{x}\pm s$). A *t*-test of independent sample data was used for pairwise comparisons, and statistical significance was evaluated with SPSS statistics 19.0 software. Values of *P* < 0.05 were considered statistically significant.

## 3. Results

### 3.1. UPLC-MS/MS Analysis of Chemical Constituents from ZBDHD

**Figure 1:**
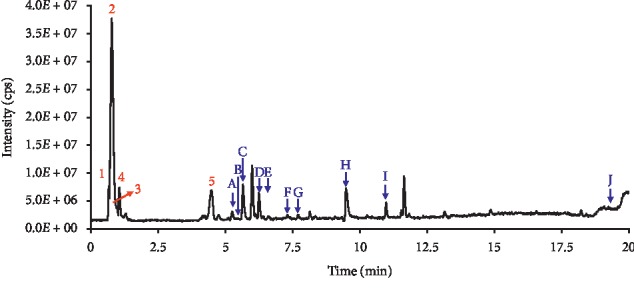
UPLC-MS/MS analysis of ZBDHD in negative ionization mode. The identified main appearing peaks with retention time between 0.5 and 12 min in total ion current (TIC) chromatogram were numbered from 1 to 5. And the specific ingredients of ZBDHD were marked from A to J. The *m*/*e*, retention time, and identification of peaks are listed in Table 2.

ZBDHD is formulated with eight herbs, which contain hundreds of components. In the present study, we analysed the components of ZBDHD using HPLC-MS/MS in both positive and negative ionization modes. The total ion current chromatogram (TIC) in negative mode is shown in Figure 1. Based on the retention time, *m*/*e*, fragmentation patterns information, and the in-house database Sciex TCM MSMS, more than 55 components were identified by using Sciex OS 1.3.1 software. Ten specific ingredients of ZBDHD were found, which were marked from A to J in Figure 1 and noted in Table 2. Most of these components were found in both modes. Berberine was, the single one, found in positive mode. We checked these components in a drug standard database https://www.drugfuture.com/standard/(in Chinese) and found out the indicate herbs (Table 2).

Additionally, the ions in appearing peaks, which were numbered from 1 to 5 in Figure 1, were also identified (Table 3). All these components were found in both modes. They are not typical markers for any herbs in ZBDHD recipe. The ions in two peaks (retention time 6.00 and 11.65 min) could not be identified because the fragmentation patterns were not discriminable.

### 3.2. ZBDHD Remedied GJD-Induced Oral Mucosa in Rat

**Figure 2:**
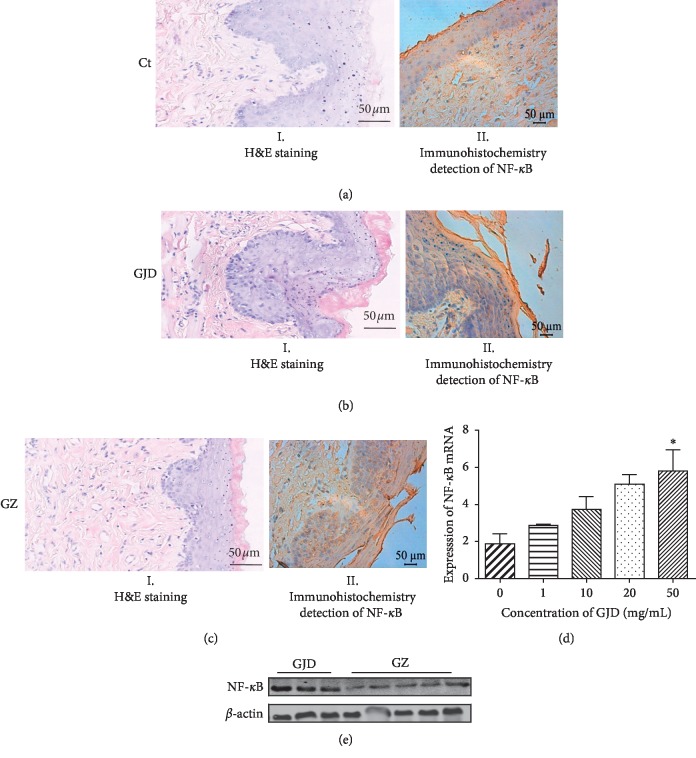
ZBDHD protected the oral mucosa in rats from GJD treatment. (I) Histological analysis of the oral mucosa. (II) Immunohistochemical staining of the oral mucosa. (a) Tissue image from a rat treated with saline (Ct group). (b) Tissue image from a rat treated with GJD (GJD). (c) Tissue image from a rat treated with ZBDHD after GJD treatment (ZBDHD). (d) The expression of NF-*κ*B in 293T, which stimulated by GJD (0, 1, 10, 20, and 50 mg/mL). (e) Expression of NF-*κ*B in rat oral mucosa of GJD and GZ groups.

The structures of oral mucosa tissue of the rats in different groups were microscopically examined. The oral mucosa of the rats in the Ct group, which has been stained with H&E, showed uniform thickness (Figure 2(a), I). In contrast, the oral mucosa of the rats in the GJD group showed obvious extensive submucosal oedema and inflammatory cell infiltration (Figure 2(b), I). The mucosal tissue of GZ group showed improvement as determined by decreased inflammatory cell infiltration (Figure 2(c), I).

The nuclear factor kappa light chain enhancer of the activated B cells (NF-*κ*B) is a ubiquitous transcription factor that is well known for its role in the innate immune response. The expression of NF-*κ*B in oral mucosa cell was immunohistochemically examined, which appears in blue colour under microscope (Figure 2, II). In contrast with Ct groups (Figure 2(a), II), the expression of NF-*κ*B in oral mucosa cell of the rats in GJD group (Figure 2(b), II) was dramatically increased. This result was validated in a GJD-treated 293T cells assay, in vitro (Figure 2(d)). In 293T cells, the transcription of NF-*κ*B gene was stimulated by GJD in a dose-dependent manner. After ZBDHD treatment for 7 days, the expression of NF-*κ*B in oral mucosa cells was improved (Figure 2(c), II). This was enhanced by Western blot (Figure 2(e)). In comparison with the expression of *β*-actin, ZBDHD treatment significantly influenced the expression of NF-*κ*B. The expression of NF-*κ*B was significantly inhibited in GZ group.

### 3.3. ZBDHD Improved GJD-Induced Metabolic Dysfunction in Rats

**Figure 3:**
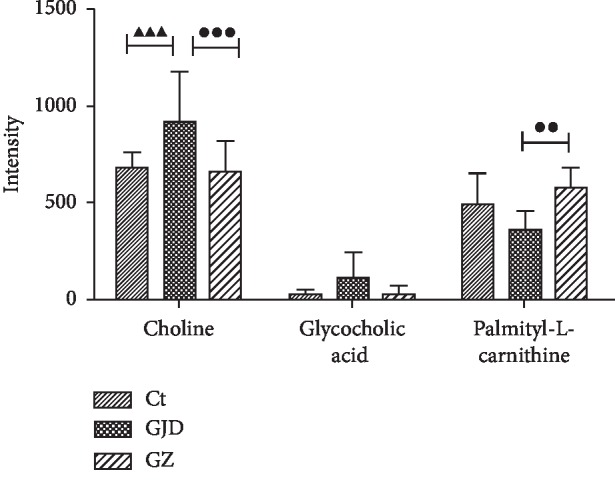
Differentially expressed metabolites in rat serum. Unitless peak heights (int.) show relative intensities in this figure. ▲▲▲ means significantly statistical difference between Ct group and Ganjiangfuzirougui decoction (GJD) group, *P* < 0.001. ● shows difference between Ganjiangfuzirougui group and Ganzhi (GJD&ZBDHD) group. ●● means statistical difference (*P* < 0.01). ●●● means significantly statistical difference, *P* < 0.001. The variable importance in the projection value (VIP, threshold > 1) of the OPLS-DA model was adopted in this project, and the *P* value of the *t*-test (*P* < 0.05) was also used to search for differentially expressed metabolites. The qualitative method to determine the differentially expressed metabolites involved searching the online database (METLIN) (to compare charge ratios determined by mass spectrometry or precise molecular mass).

The differences in the metabolomics were determined by comparing the resulting integral data derived from the spectra of the rat serum collected from three groups. Orthogonal partial least squares-discriminant analysis- (OPLS-DA-) based profiling was used to explore the intrinsic differences between the groups. When *Q*^2^ is greater than 0.4, there is a significant variation between groups [27]. The OPLS-DA results of the GJD/Ct groups and GZ/GJD are listed in Table 4. The GJD group is clearly different from the Ct and GZ groups. The concentrations of choline, glycocholic acid, and palmitoyl-L-carnitine (PALC) were significantly influenced by GJD and were restored by ZBDHD treatment (Figure 3).

### 3.4. ZBDHD Stimulated Oxidative Metabolism in Cells

**Figure 4:**
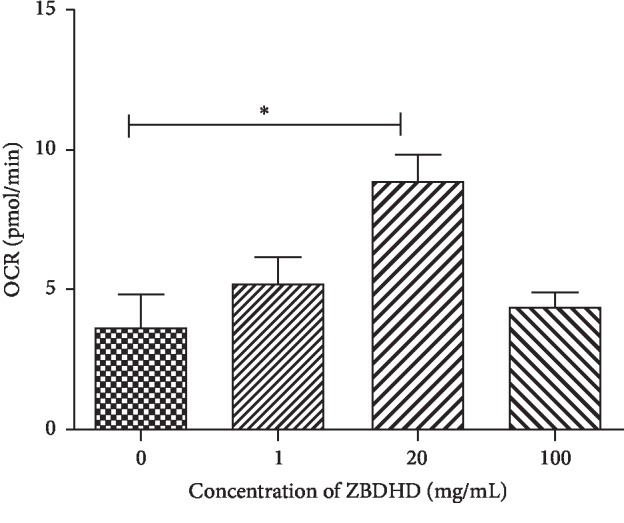
The changes of the oxygen consumption rate in 293T cells with different ZBDHD treatment. The oxygen consumption rate is presented on the *y*-axis, and the concentration of the ZBDHD is presented on the *x*-axis; values are compared against those of the 0 mg/mL group, ^*∗*^*P* < 0.05.

We hypothesized that ZBDHD would restore the concentration of the three metabolomics in GJD rats by influencing the oxidative metabolism in cells. To enhance the effects of ZBDHD, which changes the concentration levels of ATP and CuZn-SOD (Table 5) and the oxygen consumption rate (Figure 4), the cells were evaluated in vitro. The ATP concentration in the cells was increased by 100 mg/mL ZBDHD treatment. The unit concentration of CuZn-SOD in 20 mg/mL ZBDHD treated cell was significantly increased (102.33 ± 28.115 U/mL). And the concentration decreased slightly (93.04 ± 32.341 U/mL) when the ZBDHD concentration increased to 100 mg/mL.

### 3.5. ZBDHD Modulates Oxidative Metabolism through the Keap1-Nrf2-ARE Pathway

**Figure 5:**
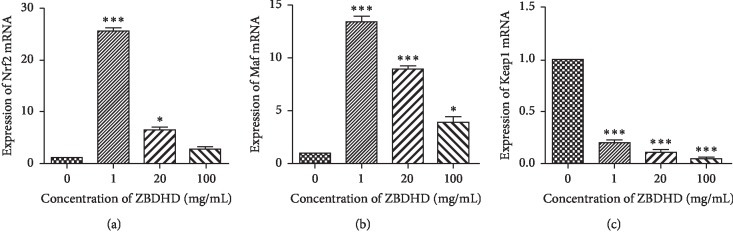
Transcription of cytokines, Nrf2, Maf, and Keap1, influenced by different concentrations of ZBDHD in 293T cells. (a) Expression of Nrf2 mRNA. (b) Expression of Maf mRNA. (c) Expression of Keap1 mRNA. The *y*-axis presents the mRNA expression level, and the *x*-axis presents the dose concentration of ZBDHD. Values are compared with those of the (ZBDHD 0 mg/mL) group, ^*∗*^*P* < 0.05, ^*∗∗*^*P* < 0.01, and ^*∗∗∗*^*P* < 0.001.

We used a Dual-Luciferase Reporter assay to elucidate the potential pathway, which is modulated by ZBDHD. If the fluorescence signal increased, then the correlated signal cytokine would be influenced by ZBDHD. Seven cytokines were tested in this study: antioxidant response element (ARE), hypoxia-inducible factor (HIF-1), cell cycle-related transcription factor response element (E2F), promoter tumour suppressor protein 53 (pP53), interferon-stimulated response element (ISRE), serum response element (SRE), sis-inducible element, and sis-inducible reaction element (SIE). The expression ratios of different cytokines in the treatment assays at different ZBDHD concentrations are listed in Table 6. Only the expression of ARE and pP53 mRNA was significantly increased by ZBDHD treatment, on the concentration of 20 mg/mL and 1 mg/mL. However, these effects were attenuated when the concentration of ZBDHD increased to 100 mg/mL and 20 mg/mL. On the contrary, the expression of HIF-1 mRNA was inhibited by ZBDHD. But this decrease was not significant. The expression of other cytokines, such as SRE, ISRE, and E2F mRNA, were also influenced by ZBDHD. But no significant signal was obtained. This result suggested that ZBDHD may modulate oxidative metabolism through the Keap1-Nrf2-ARE signalling pathway. Therefore, we demonstrated that the transcription of Nrf2 and Maf was significantly increased and Keap1 was significantly decreased by ZBDHD (Figure 5).

## 4. Discussion

“Yin” (cold) and “Yang” (fire, hot) syndrome types form the basis of TCM therapies. ZBDHD, a traditional Chinese patent medicine, is frequently given to relieve menopausal symptoms [21] and polycystic ovary syndrome hyperandrogenism [22] in patients, who present symptoms of “yin deficiency and fire exuberance” such as oral ulcers, hot flashes and night sweats. Our research suggested that ZBDHD can improve GJD-induced oral ulcers (Figure 2) in rat models. A previous study demonstrated that excessive intake of GJD can enhance oxidative stress reactions and cause a series of symptoms, oral ulcers and burning eyes, described as “Yin deficiency and fire exuberance” [25].

The capability of ZBDHD to mediate the GJD effect on the metabolites, choline, glycocholic acid, and PALC, in the serum of rats was determined by a metabolomics study (Figure 3). As is well known, choline can promote lipid metabolism [28]. Additionally, glycocholic acid is a kind of bile acid that binds to glycine to promote the production of ROS [29]. In addition, PALC, a long-chain derivative of L-carnitine, can induce the oxidative decomposition of fatty acids in mitochondria and promote lipid metabolism [30]. It also plays an important role in the development of some diseases by alleviating oxidative stress and lipid peroxidation [31–33]. Therefore, we speculate that ZBDHD can restore GJD-induced imbalance in lipid metabolism in vivo.

The oxygen consumption rate (OCR) (Figure 4) in cells was significantly increased by ZBDHD treatment (at concentrations >20 mg/mL), and ATP, produced by respiration, was increased simultaneously (Table 5). We suggest that ZBDHD influences lipid metabolism and promotes cell activity. However, the increased yield in energy production results in incomplete oxidation of O_2_, which results in the formation of ROS. “Oxidative stress,” which is caused by ROS, can significantly change the kinds and content levels of various metabolites in the body [34]. In healthy conditions, excessive ROS are decomposed by SOD in vivo to maintain the balance in the internal environment [35]. Using an in vitro assay, we evaluated the efficiency of ZBDHD in the improvement of CuZn-SOD in cells. The results suggested that ZBDHD can relieve GJD-induced oxidative stress. Is this how ZBDHD works?

ZBDHD had a significant effect on the transcription of ARE (Table 6). ARE is the master regulator of the total antioxidant system in cells, and the cytokines Nrf2 and Keap1 are involved in the activation of ARE [36, 37]. Therefore, we identified this effect with real-time RT-PCR on the ARE-related cytokines, Nrf2, Maf, and Keap1. The real-time RT-PCR results showed that ZBDHD could significantly upregulate the transcription of Nrf2 and Maf and downregulate the transcription of Keap1. It is our understanding that, in the ARE signalling pathway, Nrf2 binds to Keap1 in the cytoplasm. When Keap1 is degraded and inactivated under oxidative stress, Nrf2 is released and translocated into the nucleus, where it combines with small Maf (musculoaponeurotic fibrosarcoma) to form a Nrf2-maf heterodimer. Nrf2-maf heterodimers bind to ARE to activate the expression of ARE-dependent antioxidant genes and thus protect the body from oxidative stress [38, 39]. In contrast, GJD stimulates the expression of NF-*κ*B, another cytokine related to ARE. These results suggest that ZBDHD can enhance the Keap1-Nrf2-ARE pathway to provide antioxidants that relieve “oxidative stress.”

Identification of biologically active ingredients in medicinal hers is a major challenge for TCM research and quality control. This is because, generally, prescribed formulas in TCM involve a complex herbal system, which contains hundreds or even thousands of different chemical ingredients. Changes and losses of certain constituents may attenuate or even change its clinic efficiency. In the present study, more than 55 components in ZBDHD were identified with UPLC-MS/MS. And ten components were markers for herbs in ZBDHD recipe, but none for Fuling. Fuling (Sclerotium of *Poria cocos* (Schw.) Wolf) is a saprophytic fungus, in which the polysaccharide was considered to be the active fraction [40]. Generally, plant root contained more polysaccharides, such as Shudihuang (steamed root of the *Rehmannia glutinosa* Libosch. Preparata) and Zhimu (root of *Anemarrhena asphodeloides* Bge. is Zhimu) [19]. Therefore, we proposed the D-galactose/D-(+)-glucose/D-(+)-mannose (peak 1 in Figure 1) that could be extracted from these Fuling, Shudihuang and Zhimu. Quinic acid and chlorogenic acid (peaks 2 and 5 in Figure 1) belong to phenolic compounds, which widely exist in living creatures, especially in kingdom Plantae [41]. Citric acid and L-malic acid (peaks 3 and 4 in Figure 1) are mainly found in fruits [42]. In ZBDHD, Shanzhuyu is dried fruit of *Cornus officinalis* Sieb. Et. So, we proposed, in this study, that citric acid and L-malic acid were exacted from Shanzhuyu. In our present work, ingredients from 8 herbs in ZBDHD were found. And some of them, ions in main appearing peaks, may be the marker molecules for ZBDHD quality control.

This study found that ZBDHD can promote oxidative metabolism to prevent lipid peroxidation and inhibit oxidative stress which is induced by GJD. In vitro, ZBDHD treatment enhanced the Keap1-Nrf2-ARE signalling pathway and increased CuZn-SOD levels, which may restore the abnormal metabolic processes. The Keap1-Nrf2-ARE signalling pathway regulates the expression of a series of antioxidant enzymes including SOD, which is an important antioxidant enzyme in organisms and the primary scavenger of free radicals [43, 44]. This action may be an important mechanism by which ZBDHD ameliorates the symptoms of Yin deficiency and fire exuberance in various diseases.

## 5. Conclusion

In summary, ZBDHD is widely used in the clinic as a therapeutic agent mainly for nourishing “Yin” and reducing “Yang” (fire). “Yin” “Yang” balance can keep the human body health. Biologists apply the theory of Yin and Yang to various fields of medicine to explain and analyse the regulation of human physiological function and the relationship between pathological changes and human homeostasis. Oxidative stress can lead to the imbalance of Yin and Yang in mitochondrial function [45–47]. In the present study, ZBDHD attenuated GJD-induced “Yin deficiency and high fire” through Keap1-Nrf2-ARE signalling pathway. As we know, Nrf2 is a master regulator of cellular oxidative stress response which reacts to exogenous stimuli by translocating from the cytosol to nucleus and initiating the expression of a broad range of antioxidant-defense and cytoprotective genes [38, 39]. So, ZBDHD improves the abnormal metabolites in vivo by improving the antioxidant capacity of the cells. This may be an important mechanism of its effect on nourishing “Yin” and reducing “Yang.” And which ingredients in ZBDHD are the key activators to enhance the antioxidant stress still needs investigation. Another interesting result is that low-dose ZBDHD significantly stimulated the expression of p53. In some study, the antitumour function of p53 was compared with that of “Yin,” which can repair or remove the severely damaged cells to avoid their negative effects on health [48]. This may be another nourishing “Yin” and reducing “Yang” mechanism of ZBDHD.

## Figures and Tables

**Table 1:** Real-time PCR primer sequence.

Target cytokines	Primer	Length of target fragment (bp)
*β*-actin	F: 5′-GGAAATCGTGCGTGACAT-3′	182
	R: 5′-AGGAAGGAAGGCTGGAAGAG-3′	
Nrf2	F: 5′-TGTAAGTCCTGGTCATCGG-3′	546
	R: 5′-AAGGTCAAATCCTCCTAAATC-3′	
Maf	F: 5′-CAGGACGAGCGACCAT-3′	524
	R: 5′-GGCGGACAATAGAGGAAA-3′	
Keap1	F: 5′-ACTGTACCTGTTGAGGCACTTT-3′	193
	R: 5′-GCACATGATTCCCGCTTT-3′	

**Table 2:** Peak assignments of specific ingredients in ZBDHD via UPLC-MS/MS.

Pk. No.	RT (min)	m/e	Elemental composition	Identification	Indicate herb
A	5.26	421.08	C_19_H_18_O_11_	Mangiferin	Zhimu (*Anemarrhena asphodeloides* Bge.)
B	5.29	341.16	C_20_H_24_NO_4_	Phellodendrine	Huangbai (cortex *Phellodendron chinense* Schneid.)
C	5.66	435.15	C_17_H_26_O_10_·HCOOH	Loganin + HCOOH	Shanzhuyu (*Cornus officinalis* Sieb. et)
D	6.25	479.16	C_23_H_28_O_11_	Paeoniflorin/Albiflorin	Shaoyao (radix *Dioscorea opposita* Thunb.) and Mudanpi (*Paeonia suffruticosa* Andr.)
E	6.25	525.16	C_23_H_28_O_11_·HCOOH	Paeoniflorin + HCOOH	
F	7.31	623.20	C_29_H_36_O_15_	Verbascoside	Shudihuang (Radix *Rehmannia glutinosa* Libosch. Preparata)
G	7.745	335.12	C_20_H_18_NO_4_	Berberine	Huangbai (cortex *Phellodendron chinense* Schneid.)
H	9.49	965.50	C_45_H_76_O_19_·HCOOH	Timosaponin BII + HCOOH	Zhimu (*Anemarrhena asphodeloides* Bge.)
I	10.98	583.18	C_30_H_32_O_12_	Benzoylpaeoniflorin	Shaoyao (radix *Dioscorea opposita* Thunb.) and Mudanpi (*Paeonia suffruticosa* Andr.)
J	19.07	515.37	C_32_H_50_O_5_	Alisol B 23-acetate	Zexie (*Rhizoma Alisma orientalis*)

**Table 3:** Identified ingredients in appearing peaks.

Pk. No.	RT (min)	m/e	Elemental composition	Identification
1	0.76	179.06	C_6_H_12_O_6_	D-galactose/D-(+)-glucose/D-(+)-mannose
2	0.78	191.06	C_7_H_12_O_6_	Quinic acid
3	0.86, 0.97	113.01	C_4_H_6_O_5_	L-malic acid
4	1.06	191.02	C_6_H_8_O_7_	Citric acid
5	4.41	353.09	C_16_H_18_O_9_	Chlorogenic acid

**Table 4:** OPLS-DA score of serum comparisons among groups.

Groups	*R* ^2^ *X*	*R* ^2^ *Y*	*Q* ^2^
GJD/Ct	0.306	0.991	0.727
GZ/GJD	0.378	0.994	0.796

**Table 5:** Effect of ZBDHD on ATP and CuZn-SOD in cells ($\bar{X}+s$).

Con. of ZBDHD (mg/mL)	ATP (*μ*mol/g protein)	CuZn-SOD (U/mL)
0	5.00 ± 0.045	69.24 ± 34.002
1	5.08 ± 0.116	73.50 ± 21.495
20	5.28 ± 0.060	102.33 ± 28.115^*∗*^
100	5.48 ± 0.382^*∗∗*^	93.04 ± 32.341^*∗*^

Note: ^*∗∗*^*P* < 0.01 in comparison with that of 0 mg/mL ZBDHD.

**Table 6:** Effects of ZBDHD as shown with luciferase activity ($\bar{X}+s$).

Target cytokines	Concentration of ZBDHD (mg/mL)			
	0	1	20	100
**ARE**	**0.69** ± **0.010**	**0.72** ± **0.391**	**1.99** ± **0.377**^*∗∗*^	**1.12** ± **0.213**^*∗*^
HIF-1	0.41 ± 0.189	0.28 ± 0.047	0.27 ± 0.064	0.26 ± 0.035
E2F	0.40 ± 0.039	0.30 ± 0.022	0.79 ± 0.616	0.39 ± 0.044
pP53	0.12 ± 0.111	**1.43** ± **0.371**^*∗∗∗*^	0.20 ± 0.004	0.23 ± 0.034
ISRE	0.56 ± 0.009	0.64 ± 0.145	0.53 ± 0.078	0.30 ± 0.019
SRE	0.81 ± 0.058	0.47 ± 0.117	0.68 ± 0.067	0.21 ± 0.066
SIE	0.08 ± 0.023	0.02 ± 0.007	0.05 ± 0.033	0.09 ± 0.025

## Data Availability

All data used to support the findings of this study are included within the article and these data also can be accessible on website https://www.force11.org/article/.
